# Phononic Crystal Waveguide Transducers for Nonlinear Elastic Wave Sensing

**DOI:** 10.1038/s41598-017-14594-4

**Published:** 2017-11-07

**Authors:** Francesco Ciampa, Akash Mankar, Andrea Marini

**Affiliations:** 1 Materials and Structures Centre (MAST), Department of Mechanical Engineering, University of Bath, Claverton Down, BA2 7AY Bath, UK; 2 Escola d’Enginyeria de Telecomunicació i Aeroespacial de Castelldefels, Universitat Politècnica de Catalunya, 08860 Castelldefels (Barcelona), Spain; 3 ICFO-Institut de Ciencies Fotoniques, The Barcelona Institute of Science and Technology, 08860 Castelldefels (Barcelona), Spain

## Abstract

Second harmonic generation is one of the most sensitive and reliable nonlinear elastic signatures for micro-damage assessment. However, its detection requires powerful amplification systems generating fictitious harmonics that are difficult to discern from pure nonlinear elastic effects. Current state-of-the-art nonlinear ultrasonic methods still involve impractical solutions such as cumbersome signal calibration processes and substantial modifications of the test component in order to create material-based tunable harmonic filters. Here we propose and demonstrate a valid and sensible alternative strategy involving the development of an ultrasonic phononic crystal waveguide transducer that exhibits both single and multiple frequency stop-bands filtering out fictitious second harmonic frequencies. Remarkably, such a sensing device can be easily fabricated and integrated on the surface of the test structure without altering its mechanical and geometrical properties. The design of the phononic crystal structure is supported by a perturbative theoretical model predicting the frequency band-gaps of periodic plates with sinusoidal corrugation. We find our theoretical findings in excellent agreement with experimental testing revealing that the proposed phononic crystal waveguide transducer successfully attenuates second harmonics caused by the ultrasonic equipment, thus demonstrating its wide range of potential applications for acousto/ultrasonic material damage inspection.

## Introduction

The major difficulty in the characterisation of a material degradation process is the lack of very few measurable signs of damage prior to the onset of micro-cracks, which often precedes catastrophic failures. Nonlinear elastic wave spectroscopy (NEWS) methods have proven to be very sensitive and efficient in revealing incipient stages of damage development by measuring material nonlinear elastic effects such as harmonics (multiples) and sub-harmonics (sub-multiples) of the excitation frequency^1–3^. For example, Gun *et al*.^4^ used NEWS methods to monitor the progress of carbonation in concrete structures deposited in pre-existing voids and micro-cracks, whereas Shifeng *et al*.^5^ determined yielding of Titatinum alloy materials with second harmonic measurements. Boccardi *et al*.^6^ recently developed a nonlinear elastic multi-path reciprocal method using a sparse array of surface bonded ultrasonic transducers in order to detect and localise micro-damage in composite materials. Scalerandi^7^ defined a nonlinear elastic indicator that could be used to classify different microscopic material features such as dislocations and micro-cracks. Nonlinear elastic effects can be generally attributed to the interaction of guided Lamb waves (GLWs) with material micro-flaws, which produce clapping motion and frictional contact at crack interfaces. In micro-damaged media that manifest anharmonicity at the atomic/molecular scale such as aluminium, steel and composites, theoretical and experimental evidence indicate that the interaction of elastic waves with micro-damage can be treated as an expansion of the elastic energy as a power series with respect to the strain^8,9^. This implies that the contribution of the second harmonic amplitude is dominant with respect to other harmonics and depends quadratically over the fundamental frequency strain. The first direct consequence is that nonlinear ultrasonic testing require high input voltages (typically ranging between 50 V and 150 V) in order to highlight second harmonic effects from the background noise. However, high voltage excitation can generate nonlinear distortions in the signal amplification process, which can contaminate the recorded structural response by creating additional and fictitious harmonic frequencies that are difficult to discern from pure nonlinear elastic features. Furthermore, traditional digital filters and advanced signal processing algorithms such as phase symmetry analysis^10,11^ and the scaling subtraction method^12^ have been developed to enhance harmonics extraction. Nevertheless, signal manipulation caused by digital filtering and cumbersome signal calibration processes (e.g. phase synchronisation during continuous excitation) may alter the measured waveforms, thus severely affecting the estimation of material elastic nonlinearities.

Phononic crystals (PCs) with periodic structures are an innovative solution to the problem of filtering specific frequencies without modifying the acquired signals. In analogy to photonic crystals in optics, PCs have been studied since the pioneering work of Lord Rayleigh in 1887^13^ and a comprehensive review of the theoretical development of PCs can be found in Asfar and Nayfeh^14^ and, more recently, in Maldovan^15^. PCs are materials characterised by frequency band gaps in which GLWs propagation is effectively prohibited^16^. Outside these band gaps, elastic waves can propagate in any direction of the material so that the periodic structure itself can behave as a natural harmonic filter. Unlike acoustic metamaterials in which the band gap mechanism is similar to that of vibration absorbers^17^, band gaps in PCs are physically induced by the Bragg scattering effect, i.e. through the coupling with counter-propagating waves excited resonantly when the GLW wave-vector matches ${k}_{{\rm{B}}G}=2\pi /{\rm{\Lambda }}$ (with ${\rm{\Lambda }}$ being the wavelength of the periodic structure) and leading to destructive interference in specific frequency bands (known as stop bands). Such a wavelength ${\rm{\Lambda }}$ can be arbitrarily chosen to achieve the stop band in the desired frequency range. Depending on their geometries and mechanical constituents, PCs can be divided into three main categories: (a) materials with two or more solids of different elastic properties in a periodic repetition^18–20^, (b) materials with enclosed periodic scattering units, cavities and holes containing fluids^21–23^, and (c) mono-materials characterised by periodic corrugated waveguides^24,25^. The first two types of PCs have been extensively used in applications such as noise suppressors^16^, acoustic diodes^26^ and acoustic rectifiers^27^. Miniaci *et al*.^28^ were the first in using PC structures with cross-like holes using the Bloch-Floquet theory in order to filter out undesired frequencies on a damaged aluminium plate. They combined the filtering capabilities of PC devices and the focusing functionality of chaotic structures in order to enhance the extraction of harmonic frequencies and focus the elastic energy at the damage location using a time reversal-NEWS approach. Despite much progress in the design of PCs of types (a) and (b), substantial modifications of the host structure such as holes and inclusions are generally required for the tuning of band gaps. This could significantly affect the fabrication complexity and, ultimately, the structural integrity of components, thus preventing their use in real applications.

**Figure 1:**
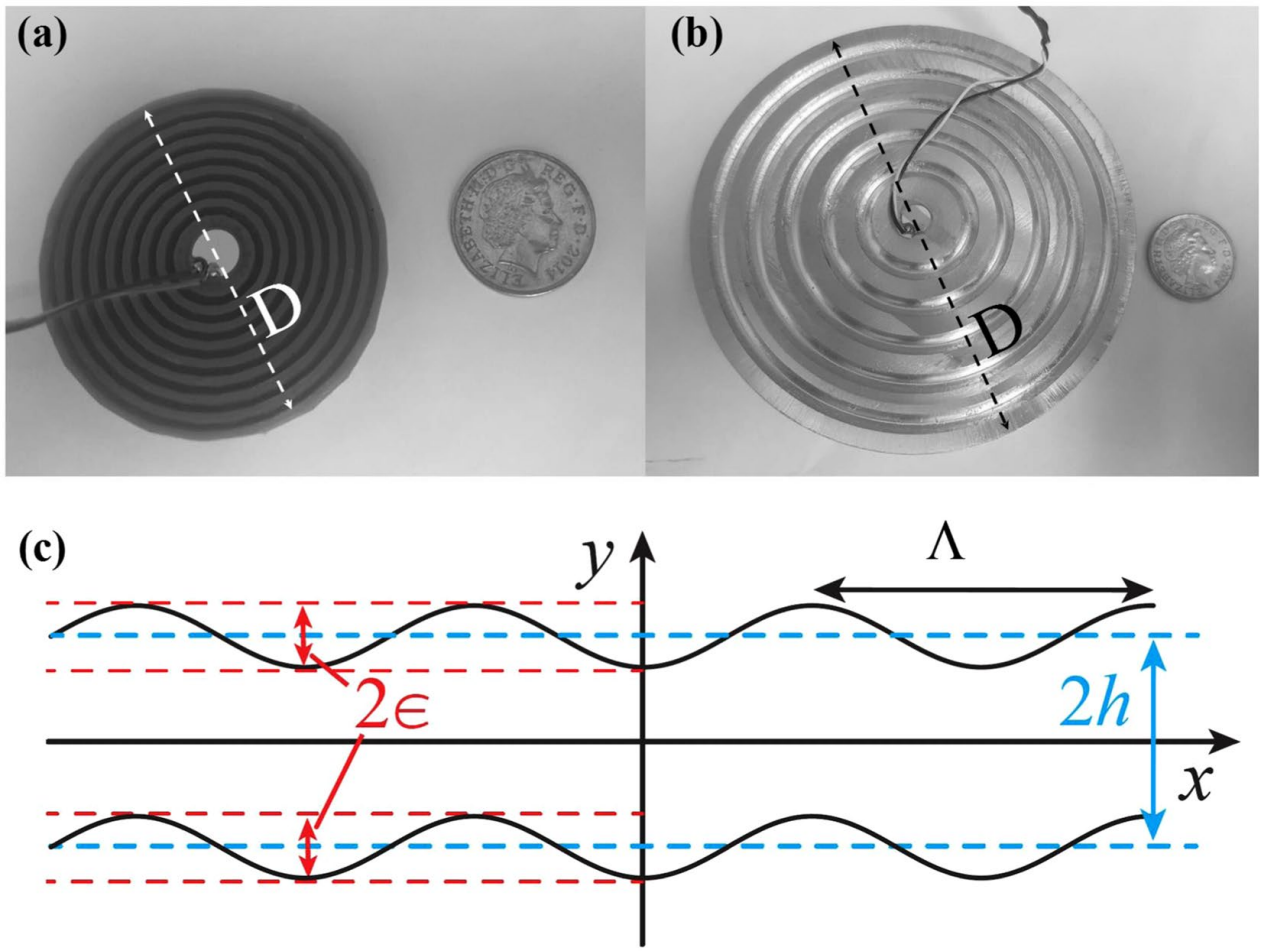
(**a**) ABS (phononic crystal) PC waveguide transducer with corrugation wavelength Λ = 2.1 mm and diameter *D* = 45.6 mm. **(b)** Aluminium PC waveguide transducer with Λ = 5.7 mm and *D* = 72 mm. **(c)** Illustration of the geometrical parameters used to design the adiabatic model for PC waveguides: 2*∈* is the corrugation depth, Λ is the corrugation wavelength at the second harmonic frequency $2{f}_{0}$, and 2*h* is the plate average thickness.

Here we overcome this limitation by developing the first prototype of nonlinear ultrasonic sensing device using PC structures with periodic waveguides and providing theoretical and experimental evidence of its functionality. In practice, such a PC transducer consists of a piezoelectric sensor bonded on a circular plate with sinusoidal corrugations that is integrated on the surface of the test component without altering its mechanical and geometrical properties. This sensing device is realized with two different materials [aluminium and acrylonitrile butadiene styrene (ABS) polymer] and it is manufactured using both 3D printing and computer numerical control (CNC) machining processes [see Fig. 1(a,b)]. The PC waveguide sensor is designed to exhibit stop bands at specific second harmonic frequencies $2{f}_{0}$ with the support of a perturbative theoretical framework describing GLW propagation in sinusoidal corrugated plates.

## Results and Discussion

### Adiabatic coupled-mode theory for the design of PC waveguide transducers

A number of researchers already studied the dispersion relations of GLWs with different periodic surface profiles^14,24,25,29^. Kundu *et al*.^30^ defined numerical solutions of Rayleigh-Lamb dispersion relations for symmetrically corrugated plates, whilst Morvan *et al*.^31^ performed numerical finite element simulations to analyse Lamb wave propagation in plates with rectangular gratings. Asfar *et al*.^32^ used a perturbation theory of the GLW problem in order to analyse the coupling of the forward unperturbed symmetric Lamb mode, ${S}_{0}^{+}$, with the backward antisymmetric, ${A}_{0}^{-}$, in plates with finite corrugations.

We here develop a perturbative coupled-mode theory based on GLW propagation in order to identify band gaps in PC waveguides as function of material properties and geometrical features such as the corrugation depth 2*∈*, the average plate thickness 2*h*, and the corrugation period Λ and distance *D* [see Fig. 1(c)]. Owing to the periodic corrugation, frequency stop bands arise on the edges of the first Brillouin zone ($\pm \pi /{\rm{\Lambda }}$) for both aluminium ($E\,=\,70$ GPa, $\nu \,=\,0.33$ and $\rho \,=\,2700$ kg/m^3^) and ABS PC waveguides ($E\,=\,2.3$ GPa, $\nu \,=\,0.35$ and $\rho \,=\,1041$ kg/m^3^), with *E* being the Young’s modulus, $\nu$ the Poisson’s ratio and $\rho$ the density. We start our theoretical analysis from the most common approach for solving the GLW problem in bounded isotropic media by applying the “potentials method” (also known as Helmholtz decomposition^33^) to the invariant form of Navier’s equations in absence of corrugation (i.e. when 2*∈* = 0). Assuming harmonic waves with frequency $f$ propagating in the *x*-direction and bounded by planes ${y}_{\pm }=\pm h$, two well-known independent dispersion relations for the symmetric ${k}_{{\rm{S}}}(f)$ and the anti-symmetric ${k}_{{\rm{A}}}(f)$ wavenumbers are obtained. Thus, moving to the PC corrugated waveguide, we assume that the unperturbed lowest symmetric ${S}_{0}$ and antisymmetric ${A}_{0}$ modes follow adiabatically the periodic corrugation such that their displacement vector mode profiles are ${{\boldsymbol{u}}}_{{\rm{S}},A}(\tilde{y})$, where $\tilde{y}=y-\epsilon \,\cos \,\mathrm{(2}\pi x/{\rm{\Lambda }})$, so that we can study the coupling of such unperturbed modes induced by the corrugation itself. Thus, we set the Ansatz for the displacement ${\bf{u}}(x,y)$ as the superposition of both forward and backward symmetric and antisymmetric waves

1 $$\begin{array}{rcl}{\bf{u}}(x,y) & = & \mathrm{Re}\{[{a}_{{\rm{S}}}^{+}(x){{\bf{u}}}_{{\rm{S}}}^{+}(\tilde{y}){{\rm{e}}}^{{\rm{i}}{k}_{{\rm{S}}}x}+{a}_{{\rm{S}}}^{-}(x){{\bf{u}}}_{{\rm{S}}}^{-}(\tilde{y}){{\rm{e}}}^{-{\rm{i}}{k}_{{\rm{S}}}x}+\\  &  & +{a}_{{\rm{A}}}^{+}(x){{\bf{u}}}_{{\rm{A}}}^{+}(\tilde{y}){{\rm{e}}}^{{\rm{i}}{k}_{{\rm{A}}}x}+{a}_{{\rm{A}}}^{-}(x){{\bf{u}}}_{{\rm{A}}}^{-}(\tilde{y}){{\rm{e}}}^{-{\rm{i}}{k}_{{\rm{A}}}x}]{{\rm{e}}}^{-2{\rm{i}}\pi ft}\},\end{array}$$

where ${a}_{{\rm{S}},{\rm{A}}}^{\pm }$ are the amplitudes of the modes, and the signs + and − denote forward and backward GLWs. In order to phenomenologically study the effect of periodic corrugation, the general solution (1) is inserted into Navier’s equation and expanded in multiple scales at first order (see the Supplementary Material for more detailed derivation). 1The solvability condition of the following vectorial system of equations is obtained by taking the scalar product with the symmetric and antisymmetric forward and backward unperturbed modes. We find that the couplings between forward and backward modes of identical symmetry vanish, while couplings between forward and backward modes of opposite symmetries remain finite. Hence, in agreement with the Bragg mechanism, the destructive interference of scattered Lamb modes induced by the corrugation gives rise to new dispersion equations for forward propagating modes

2 $$\frac{{d}^{2}{a}_{{\rm{S}},{\rm{A}}}^{+}(x)}{d{x}^{2}}={\rm{i}}\,[\frac{2\pi }{{\rm{\Lambda }}}-({k}_{{\rm{A}}}+{k}_{{\rm{S}}})]\frac{d{a}_{{\rm{S}},{\rm{A}}}^{+}(x)}{dx}+{C}_{S}{C}_{A}{(\frac{\pi \epsilon }{{\rm{\Lambda }}})}^{2}{a}_{{\rm{S}},A}^{+}(x),$$

which provide the solutions ${a}_{{\rm{S}},{\rm{A}}}^{+}(x)={A}_{{\rm{S}},{\rm{A}}}^{+}{{\rm{e}}}^{{\rm{i}}\beta x}$, where the wavenumber correction $\beta$ induced by the corrugation is

3 $$\beta =\frac{1}{2}[\frac{2\pi }{{\rm{\Lambda }}}-({k}_{{\rm{A}}}+{k}_{{\rm{S}}})]+\frac{1}{2}\sqrt{{[\frac{2\pi }{{\rm{\Lambda }}}-({k}_{{\rm{A}}}+{k}_{{\rm{S}}})]}^{2}-4{C}_{{\rm{A}}}{C}_{{\rm{S}}}{(\frac{\pi \epsilon }{{\rm{\Lambda }}})}^{2}}\mathrm{.}$$

**Figure 2:**
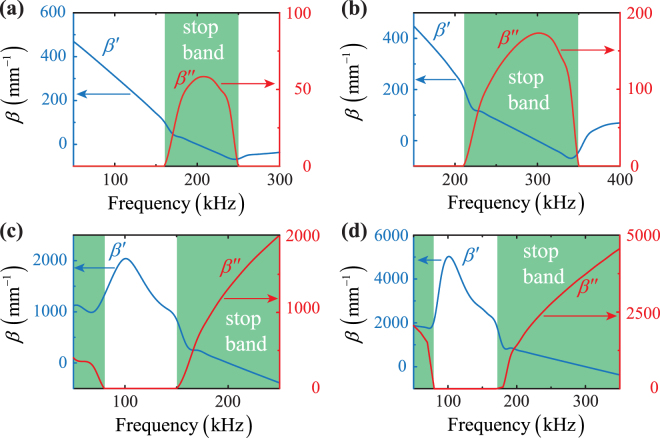
Analytical results of real (blue lines) and imaginary (red lines) parts of the corrugation-induced wavevector shift $\beta (f)$ for PC waveguides with $2h$ = 10 mm and 2*∈* = 3 mm. **(a)** Aluminium PC plate with ${\rm{\Lambda }}$ = 8.1 mm ($2{f}_{0}$ = 200 kHz). (**b**) Aluminium PC plate with ${\rm{\Lambda }}$ = 5.7 mm ($2{f}_{0}$ = 300 kHz). (**c**) ABS PC plate with ${\rm{\Lambda }}$ = 2.1 mm ($2{f}_{0}$ = 200 kHz). **(d)** ABS PC plate with ${\rm{\Lambda }}$ = 1.4 mm ($2{f}_{0}$ = 300 kHz).

The amplitudes ${A}_{{\rm{S}},A}^{+}$ are constants, whilst terms ${C}_{{\rm{S}},{\rm{A}}}$ in Eqs (2) and (3) are functions of the material and geometric features of the PC waveguide (their full integral expressions are provided in the supplementary material). In order to obtain stop bands in the desired second harmonic frequency range, both wavenumbers ${k}_{{\rm{S}},{\rm{A}}}$ from the unperturbed GLW problem are set equal to ${k}_{{\rm{S}},{\rm{A}}}\mathrm{(2}{f}_{0})$ and the corrugation period to ${\rm{\Lambda }}\mathrm{(2}{f}_{0})\,=\,2\pi /[{k}_{{\rm{S}}}\mathrm{(2}{f}_{0})+{k}_{{\rm{A}}}\mathrm{(2}{f}_{0})]$. Band gaps are analytically estimated by analysing the wave extinction dependency over the frequency through the imaginary part of $\beta (f)=\beta ^{\prime} (f)+{\rm{i}}\beta ^{\prime\prime} (f)$. Figure 2 depicts the analytical results of both real and imaginary parts of the corrugation-induced wavevector shift $\beta (f)$ in both aluminium and ABS PC waveguides by setting $2h$ = 10 mm and 2*∈* = 3 mm. Note that, for two spatial periods ${\rm{\Lambda }}$ = 8.1 mm ($2{f}_{0}$ = 200 kHz) and ${\rm{\Lambda }}$ = 5.7 mm ($2{f}_{0}$ = 300 kHz), the aluminium PC transducer exhibits a single stop band ranging between 160 kHz and 250 kHz [highlighted in green in Fig. 2(a)] and between 210 kHz and 350 kHz [highlighted in green in Fig. 2(b)], respectively. In particular, $\beta ^{\prime} (f)$ vanishes at the second harmonic frequency in both aluminium PC plates while $\beta ^{\prime\prime} (f)$ becomes maximum, thus suppressing propagation into evanescent waves in such a frequency range. The ABS corrugated structure, instead, displays two stop band regions for each wavelength ${\rm{\Lambda }}$. Specifically, for ${\rm{\Lambda }}$ = 2.1 mm ($2{f}_{0}$ = 200 kHz), stop bands are obtained at frequencies $f\, < \,80$ kHz and $f\, > \,150$ kHz [highlighted in green in Fig. 2(c)], whereas for ${\rm{\Lambda }}$ = 1.4 mm ($2{f}_{0}$ = 300 kHz), stop bands are achieved at $f\, < \,80$ kHz and $f\, > \,180$ kHz [highlighted in green in Fig. 2(d)]. Similarly to the aluminium case, $\beta ^{\prime} (f)$ vanishes at the second harmonic frequency, where $\beta ^{\prime\prime} (f)$ is maximum.

**Figure 3:**
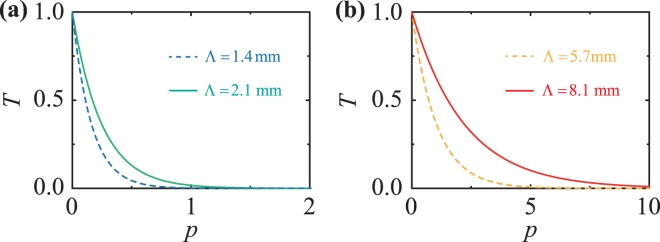
Analytical results of the transducer transmission coefficient $T$. **(a)** ABS PC waveguides with ${\rm{\Lambda }}$ = 2.1 mm (green continuous line) and ${\rm{\Lambda }}$ = 1.4 mm (blue dashed line). **(b)** Aluminium PC waveguides with ${\rm{\Lambda }}$ = 8.1 mm (red continuous line) and ${\rm{\Lambda }}$ = 5.7 mm (yellow dashed line).

Another key parameter to be considered for the design of PC structures is the corrugation distance $P=p{\rm{\Lambda }}$, with $p$ an integer number. In particular, the transmission coefficient $T=\exp [-\beta ^{\prime\prime} (f)P]\ll 1$ provides the distance required for the wave intensity to be attenuated. Such information is necessary to evaluate the number of corrugations upon which the stop band region becomes effective. As illustrated in Fig. 3(a), $p$ = 10 and $p$ = 5 periods of corrugation are required for the aluminium PC waveguides with ${\rm{\Lambda }}$ = 8.1 mm and ${\rm{\Lambda }}$ = 5.7 mm, respectively. Conversely, only $p$ = 2 periods are sufficient for both ABS PCs with ${\rm{\Lambda }}$ = 2.1 mm and ${\rm{\Lambda }}$ = 1.4 mm to attenuate nearly 100% of the elastic energy at the stop band. The transmission coefficient $T$ is proportional to the wavelength ${\rm{\Lambda }}$ of the corrugated waveguide, i.e. it is inversely proportional to the sum of the symmetric, ${k}_{S}$, and antisymmetric, ${k}_{A}$, wavenumbers, which are, in turn, dependent on the material properties. Hence, a shorter wavelength ${\rm{\Lambda }}$ would generally imply less periods $p$ sufficient to attenuate most of the elastic energy at the band gap. This was the case of the ABS PC structure compared to the aluminium one, due to the lower values of the Young’s modulus and density. In this work, both isotropic materials were selected as they are lightweight, easy to machine (using 3D printing and CNC processes) and relatively cheap. From our perspective, other materials such as aluminium alloys and polymers (e.g. both thermoplastic and thermosetting polymers) could in principle be used, although the overall weight and fabrication costs of the PC sensing device may increase considerably. Finally we note that, although the theory presented here is 1 + 1 dimensional, it provides approximate analytical expressions for a rapid identification of the frequency stop bands because we focus on the frequency range where only the fundamental ${A}_{0}$ and ${S}_{0}$ modes exist and further bands arising from the coupling of different uncorrugated modes are not allowed in this frequency range.

### NEWS ultrasonic testing with PC waveguide transducers

Based on the results of the adiabatic model, four PC sensing devices were designed and manufactured to naturally filter out specific second harmonic frequencies. Their geometrical properties are reported in Table 1 and two examples with ABS (${\rm{\Lambda }}$ = 2.1 mm) and aluminium (${\rm{\Lambda }}$ = 5.7 mm) PCs transducers are illustrated in Fig. 1(a,b), respectively.

**Table 1:** Geometrical parameters of the fabricated PC waveguide transducers.

	Aluminium		ABS	
Fundamental frequency, ${{\boldsymbol{f}}}_{{\bf{0}}}$ [kHz]	100	150	100	150
Second harmonic frequency, ${\bf{2}}{{\boldsymbol{f}}}_{{\bf{0}}}$ [kHz]	200	300	200	300
Thickness, ${\bf{2}}{\boldsymbol{h}}$ [mm]	10	10	10	10
Corrugation Depth, ${\bf{2}}{\boldsymbol{\varepsilon }}$ [mm]	3	3	3	3
Wavelength at ${\bf{2}}{{\boldsymbol{f}}}_{{\bf{0}}}$, ${\boldsymbol{\Lambda }}$ [mm]	8.1	5.7	2.1	1.4
Corrugation Distance, ${\boldsymbol{P}}$ [mm]	81 (10 ${\rm{\Lambda }}$)	28.5 (5 ${\rm{\Lambda }}$)	16.8 (8 ${\rm{\Lambda }}$)	11.2 (8 ${\rm{\Lambda }}$)
Diameter, ***D*** [mm]	96	72	45.6	34.4

**Figure 4:**
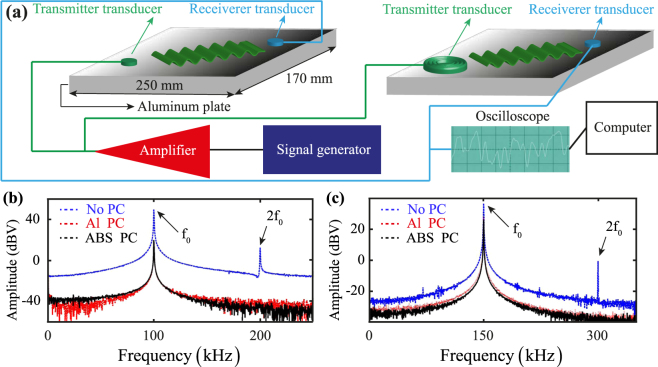
**(a)** Schematic illustration of the experimental setup for GLWs propagation on an aluminium plate-like sample. The PC waveguide and a surface bonded piezoelectric transducer are used as ultrasonic wave transmitters, whilst a second piezoelectric sensor is employed to acquire the material nonlinear elastic response. Whilst the transmitter transducers are connected to a signal generator and an amplifier, the receiver sensors are linked to an oscilloscope and a computer to process the measured waveforms. **(b,c)** Experimental material response measured by the receiver sensor using an amplified input voltage of 125 V and a driving frequency **(b)** ${f}_{0}$ = 100 kHz and **(c)** ${f}_{0}$ = 150 kHz. In both figures, the blue dashed line is the waveform acquired with no transmitter PC waveguides, whereas the dashed red and continuous black lines are the signals recorded using the aluminium and ABS PC sensing devices, respectively.

NEWS ultrasonic experiments were performed to validate the adiabatic model and to analyse the characteristics of propagating GLWs. Single tone-burst periodic signals in the $0\, < \,{f}_{0}\, < \,400$ kHz frequency range were transmitted from the piezoelectric transducer surface bonded on the PC waveguide (either aluminium or ABS) that, in turn, was glued on a homogeneous aluminium plate. Such a frequency range of interest was specifically chosen in order to generate fundamental Lamb modes ${S}_{0}$ and ${A}_{0}$ in the PC structure, so to activate the periodicity effect of band gaps induced by destructive interference only among these two waves. In order to measure the nonlinear second harmonic elastic response generated by the amplification system, a receiver piezoelectric transducer was located at direct contact with the aluminium plate ~150 mm far away from the input elastic source. We also performed the same experiments in the absence of corrugated waveguides in order to compare the strength of the recorded signals and evaluate the extinction efficiency of the proposed PC transducers. Figure 4(a) illustrates the experimental setup for the NEWS experiments with and without PC waveguide transmitter sensors. Figure 4(b,c) show the ultrasonic results for ${f}_{0}$ equal to 100 kHz and 150 kHz, respectively, at the input voltage of 125 V. In comparison with the case of the transmitter transducer directly in contact with the host aluminium plate, the elastic wave energy of received signals was attenuated in the PC sensing device, with an amplitude loss of ~25 dBV [Fig. 4(c)] at ${f}_{0}$ = 100 kHz and ~16 dBV at ${f}_{0}$ = 150 kHz [Fig. 4(c)]. However, the amplification system with no PC waveguides was not subject to any filtering and created undesired second harmonics at both driving frequencies [see dashed blue lines in Fig. 4(b,c)]. The same frequencies were, instead, successfully filtered out by using both aluminium and ABS PC waveguides (see dashed red and continuous black lines).

**Figure 5:**
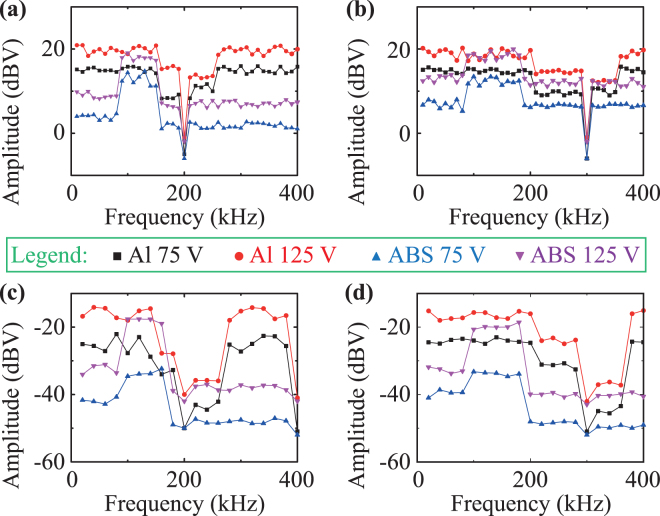
Experimental maximum amplitudes at the input voltage of 75 V and 125 V. **(a**,**b)** Peak amplitudes at the fundamental frequency using **(a)** the aluminium PC transducer with ${\rm{\Lambda }}$ = 8.1 mm and the ABS one with ${\rm{\Lambda }}$ = 2.1 mm, and using **(b)** the aluminium PC transducer with ${\rm{\Lambda }}$ = 5.7 mm and the ABS one with ${\rm{\Lambda }}$ = 1.4 mm. **(c**,**d)** Peak amplitudes the second harmonic frequency using **(c)** the aluminium PC transducer with ${\rm{\Lambda }}$ = 8.1 mm and the ABS one with ${\rm{\Lambda }}$ = 2.1 mm, and using **(d)** the aluminium PC transducer with ${\rm{\Lambda }}$ = 5.7 mm and the ABS one with ${\rm{\Lambda }}$ = 1.4 mm. In every figure, the black square and blue triangle markers indicate the aluminium and ABS PC waveguides at 75 V, whereas the red circle and purple inverted triangle markers correspond to the aluminium and ABS PC structures at 125 V.

In order to further investigate the band-gaps of PC waveguides, we experimentally recorded the peak amplitudes of both fundamental and second harmonic frequencies at two input voltages of 75 V and 125 V. The results are shown in Fig. 5 for the four sensing devices reported in Table 1. Particularly, Fig. 5(a,b) report the maximum amplitudes of fundamental frequencies using both aluminium and ABS PC waveguide transducers, whilst Fig. 5(c,d) show the measured second harmonic peak amplitudes. As expected by our theoretical calculations, whilst PC transducers do not alter fundamental and second harmonic frequencies outside the stop band region, the lowest voltages are achieved at 200 kHz and 300 kHz for both fundamental and second harmonic amplitudes, with values respectively of ~−6 dBV and ~−50 dBV. Moreover, in agreement with the adiabatic coupled-mode theory, the elastic wave extinction produces a visible stop band region in the aluminium PC waveguides ranging between ~148 kHz and ~252 kHz [Fig. 5(a–c)], and between ~198 kHz and ~345 kHz [Fig. 5(b–d)]. Similarly, two stop bands below ~78 kHz and above ~147 kHz [Fig. 5(a–c)], and below ~78 kHz and above ~182 kHz [Fig. 5(b–d)] were obtained for the ABS PCs. Such experimental results are all consistent with the predicted energy band-gaps illustrated in Fig. 2. Thus, we envisage that the proposed PC waveguide transducers are promising tools for harmonics filtering enabling a further enhancement of the accuracy and sensitivity of NEWS methods.

## Conclusions

In summary, we develop a theoretical framework for the design of PC waveguide transducers and we experimentally demonstrate that they are able to filter out undesired second order nonlinear elastic effects in NEWS experiments. These sensing devices exhibit stop bands at specific second harmonic frequencies and their design is provided by an adiabatic coupled-mode theory based on GLWs propagation in PC structures with sinusoidal corrugation. The analytical model reveals that the corrugation wavelength ${\rm{\Lambda }}$ is inversely proportional to the sum of the lowest symmetric ${k}_{{\rm{S}}}\mathrm{(2}{f}_{0})$ and antisymmetric ${k}_{{\rm{A}}}\mathrm{(2}{f}_{0})$ Lamb wavenumbers. By analysing the frequency dispersion of the corrugation-induced wavevector shift $\beta$, we design both single and dual stop bands by simply changing the PC material (ABS and aluminum). NEWS experiments confirm these theoretical predictions and indicate that both aluminium and ABS PC waveguide sensing devices enable an efficient filtering of second harmonics caused by the signal amplification system on an aluminium test sample. It should be noted that although experiments were conducted on an isotropic structure, the proposed PC transducers can be also used in orthotropic and anisotropic media. Furthermore, given the damage frequency-selective nature of NEWS methods, future work is ongoing to improve the design of PC waveguides by exploiting their multiple band gap characteristics. Finally, since PC waveguide transducers are easy to manufacture and do not require any modification of the host material, we envisage that their use can dramatically enhance the sensitivity to micro-defects of NEWS methods for various applications (e.g. aerospace, civil and nuclear) and different damage scenarios.

## Methods

### Fabrication of PC waveguide transducers

The geometrical properties of the manufactured PC structures were determined by the theoretical predicions and are reported in Table 1. Whilst ABS PC structures with sinusoidal corrugations were fabricated using conventional fused deposition modelling (FDM) 3D printing, a traditional CNC machining process was used for the aluminium PC waveguides.

### Experimental Set-up

As shown in Figs 4 and 5, 20 cycles long sine wave bursts $s(t)={A}_{0}H(t)sin\mathrm{(2}\pi {f}_{0}t)$ enclosed in a Hanning window $H(t)$, with ${A}_{0}$ being the input amplitude, were transmitted at frequencies ${f}_{0}$ ranging between 0 and 400 kHz by the piezoelectric transducer (from AmericanPiezo, with central frequency of 330 kHz, radius of 3 mm and thickness of 1 mm), which was surface bonded on the PC waveguide. Such a sensor was driven by an arbitrary waveform signal generator (TTI 50 MHz Pulse Generator T6501) connected to an amplifier (Falco Systems DC 5 MHz High Voltage WMA-300). The receiver transducer was glued directly on the aluminium plate (with dimensions of 250 × 170 × 7 mm) and it was linked to an oscilloscope (Pico Technology oscilloscope 100 V max input, Model 4424) and a personal computer in order to display the recorded waveforms. Signals received by the piezoelectric sensor were sampled at 2 MHz with an acquisition window $\tau$ = 5 ms and processed using MATLAB software. Time histories were averaged 20 times for each input frequency in order to improve the signal-to-noise ratio of measured signals. Additionally, the host aluminium sample was positioned along the edges on four foam pieces in order to reduce environmental noise effects.

## Electronic supplementary material

Supplementary Material

## Footnotes

1 See Supplemental material for further details about the adiabatic model for phononic crystal waveguides with periodic corrugation.

## References

[1] Meo M, Polimeno U, Zumpano G (2008). Detecting damage in composite material using nonlinear elastic wave spectroscopy methods. Applied Composite Materials.

[2] Ulrich T, Johnson PA, Guyer RA (2007). Interaction dynamics of elastic waves with a complex nonlinear scatterer through the use of a time reversal mirror. Physical review letters.

[3] Ostrovsky L, Johnson P (2001). Dynamic nonlinear elasticity in geomaterials. Rivista del nuovo cimento.

[4] Gun K (2016). Quantitative evaluation of carbonation in concrete using nonlinear ultrasound. Materials and Structures.

[5] Shifeng G (2016). Method and analysis for determining yielding of titanium alloy with nonlinear Rayleigh surface waves. Materials Science and Engineering: A.

[6] Boccardi S, Callá DB, Ciampa F, Meo M (2018). Nonlinear Elastic Multi-Path Reciprocal Method for Damage Localisation in Composite Materials. Ultrasonics.

[7] Scalerandi M (2016). Power laws and elastic nonlinearity in materials with complex microstructure. Physics Letters A.

[8] Delsanto, P. P. Universality of nonclassical nonlinearity. (Springer, Berlin, 2006).

[9] Landau, L. D., Lifshitz, E. M. Theory of Elasticity, Chap. III (Pergamon, Oxford, 1986).

[10] Ciampa F, Meo M (2012). Nonlinear elastic imaging using reciprocal time reversal and third order symmetry analysis. The Journal of the Acoustical Society of America.

[11] Ciampa F, Scarselli G, Meo M (2015). Nonlinear imaging method using second order phase symmetry analysis and inverse filtering. Journal of Nondestructive Evaluation.

[12] Bruno CLE, Gliozzi AS, Scalerandi M, Antonaci P (2009). Analysis of elastic nonlinearity using the scaling subtraction method. Physical Review B.

[13] Rayleigh L (1887). On the maintenance of vibrations by forces of double frequency, and on the propagation of waves through a medium endowed with a periodic structure. The London, Edinburgh, and Dublin Philosophical Magazine and Journal of Science.

[14] Asfar O, Nayfeh A (1983). The application of the method of multiple scales to wave propagation in periodic structures. SIAM Review.

[15] Maldovan M (2013). Sound and heat revolutions in phononics. Nature.

[16] Kushwaha MS, Halevi P, Dobrzynski L, Djafari-Rouhani B (1993). Acoustic band structure of periodic elastic composites. Physical review letters.

[17] Liu Z (2000). Locally resonant sonic materials. Science.

[18] Khelif A, Aoubiza B, Mohammadi S, Adibi A, Laude V (2006). Complete band gaps in two-dimensional phononic crystal slabs. Physical Review E.

[19] Hsu J-C, Wu T-T (2007). Lamb waves in binary locally resonant phononic plates with two-dimensional lattices. Applied physics letters.

[20] Hsu J-C, Wu T-T (2006). Efficient formulation for band-structure calculations of two-dimensional phononic-crystal plates. Physical Review B.

[21] Sik Ma,P, Eui Kwon Y, Young Kim Y (2013). Wave dispersion tailoring in an elastic waveguide by phononic crystals. Applied physics letters.

[22] Wang Y-F, Wang Y-S (2013). Complete bandgaps in two-dimensional phononic crystal slabs with resonators. Journal of Applied Physics.

[23] De Espinosa FM, Jimenez E, Torres M (1998). Ultrasonic band gap in a periodic two-dimensional composite. Physical Review Letters.

[24] Sorokin VS (2016). Effects of corrugation shape on frequency band-gaps for longitudinal wave motion in a periodic elastic layer. The Journal of the Acoustical Society of America.

[25] Tao, Z.-Y. and Fan, Y.-X. Orthogonality breaking induces extraordinary single-mode transparency in an elaborate waveguide with wall corrugations. *Scientific reports*, **4** (2014). 10.1038/srep07092 PMC4235311 25403089

[26] Liang B, Yuan B, Cheng J-C (2009). Acoustic diode: Rectification of acoustic energy flux in one-dimensional systems. Physical review letters.

[27] Liang B, Guo XS, Tu J, Zhang D, Cheng JC (2010). An acoustic rectifier. Nature Materials.

[28] Miniaci M (2017). Proof of concept for an ultrasensitive technique to detect and localize sources of elastic nonlinearity using phononic crystals. Physical Review Letters.

[29] Sandström SE (1986). Stopbands in a corrugated parallel plate waveguide. The Journal of the Acoustical Society of America.

[30] Kundu T, Banerjee S, Jata KV (2006). An experimental investigation of guided wave propagation in corrugated plates showing stop bands and pass bands. The Journal of the Acoustical Society of America.

[31] Morvan B, Hladky-Hennion AC, Leduc D, Izbicki JL (2007). Ultrasonic guided waves on a periodical grating: Coupled modes in the first Brillouin zone. Journal of applied physics.

[32] Asfar O, Hawwa M, Bavencoffe M, Morvan B, Izbicki JL (2013). A multiple-scale perturbation approach to mode coupling in periodic plates. IEEE transactions on ultrasonics, ferroelectrics, and frequency control.

[33] Viktorov, I.A. Rayleigh and Lamb Waves: Physical Theory and Applications (New York, Plenum press, 1967).

