# Supplementary material for: Phononic Crystal Waveguide Transducers for Nonlinear Elastic Wave Sensing
Source: Sci Rep. 2017 Nov 7;7:14712. doi: 10.1038/s41598-017-14594-4 (PMC5676704; doi:10.1038/s41598-017-14594-4)
Supplement: Supplementary file 1 — Supplementary Material [file 41598_2017_14594_MOESM1_ESM.pdf]

SUPPLEMENTARY MATERIAL
for
Phononic Crystal Waveguide Transducers for Nonlinear Elastic Wave Sensing

Francesco Ciampa
*Materials and Structures Centre (MAST), Department of Mechanical Engineering, University of Bath, Claverton Down, BA2 7AY, Bath, UK**

Akash Mankar
Escola d'Enginyeria de Telecomunicació i Aeroespacial de Castelldefels, Universitat Politècnica de Catalunya, 08860 Castelldefels (Barcelona), Spain

Andrea Marini
ICFO-Institut de Ciencies Fotoniques, The Barcelona Institute of Science and Technology, 08860 Castelldefels (Barcelona), Spain

(Dated: October 2, 2017)

Here we provide additional information on technical aspects of the adiabatic coupled-mode theory applied to the guided Lamb waves (GLW) problem in phononic crystals (PC) waveguides for the identification of stop band frequencies induced by the corrugation. As described in the main paper, the results of this theory are used for the design of PC waveguide transducers.

I. BOUNDED GUIDED LAMB WAVE PROBLEM

The most common approach for solving the GLWs problem in isotropic bodies derives from the potential methods (Helmholtz decomposition) applied to the Navier's equation in the absence of body forces [1]

$$\rho \frac{\partial^2 u_i}{\partial t^2} = (\lambda + \mu) \frac{\partial^2 u_k}{\partial d_i \partial d_k} + \mu \frac{\partial^2 u_i}{\partial d_k^2}, \tag{1}$$

where $u_i = (u_x, u_y)$ is the two-dimensional displacement vector in a Cartesian reference frame, $d_i = (x, y)$ is the coordinates vector, ρ is the density and λ and μ are the Lame' parameters, which are a combination of the Young's modulus E and the Poisson's ratio ν of the isotropic material. As in our calculations we assume a corrugated waveguide homogeneous over the z-direction, in Eq.(1) we set $u_z = 0$ and $\partial()/\partial(z) = 0$. Both Lame' parameters relate the first spatial derivative of the displacement u_i with the stress tensor σ_{ij} through the well-known Hooke's law as follows

$$\sigma_{ij} = \lambda \frac{\partial u_k}{\partial d_k} \delta_{ij} + \mu \left(\frac{\partial u_i}{\partial d_j} + \frac{\partial u_j}{\partial d_i} \right), \tag{2}$$

with δ_{ij} being the Kronecker delta. In vector form, the time harmonic displacement solution $\mathbf{u}(x, y)$ of Eq. (1) can be written as the sum of the gradient of a scalar potential Φ and the curl of a vector potential $\mathbf{\Psi}$ as follows [2]

$$\mathbf{u}(x, y) = \nabla \Phi + \nabla \times \mathbf{\Psi}. \tag{3}$$

*Electronic address: f.ciampa@bath.ac.uk

By inserting Eq. (3) into both Eq. (1) and Eq. (2), the components u_x and u_y of the displacement $\mathbf{u}(x,y)$ and the components σ_{xx}, σ_{yy} and σ_{xy} of the stress tensor can be expressed in terms of the potentials Φ and $\mathbf{\Psi} = \Psi\hat{z}$ as follows

$$\begin{aligned} u_x &= \frac{\partial \Phi}{\partial x} + \frac{\partial \Psi}{\partial y}, \\ u_y &= \frac{\partial \Phi}{\partial y} - \frac{\partial \Psi}{\partial x}, \\ \sigma_{xx} &= (\lambda + 2\mu)\frac{\partial^2 \Phi}{\partial x^2} + \lambda \frac{\partial^2 \Phi}{\partial y^2} + 2\mu \frac{\partial^2 \Psi}{\partial x \partial y}, \\ \sigma_{xx} &= (\lambda + 2\mu)\frac{\partial^2 \Phi}{\partial y^2} + \lambda \frac{\partial^2 \Phi}{\partial x^2} - 2\mu \frac{\partial^2 \Psi}{\partial x \partial y}, \\ \sigma_{xy} &= \mu \left(\frac{\partial^2 \Psi}{\partial y^2} - \frac{\partial^2 \Psi}{\partial x^2} + 2\frac{\partial^2 \Phi}{\partial x \partial y} \right), \end{aligned} \tag{4}$$

where $\hat{z}$ is the unit vector over the z-direction. Assuming a harmonic wave with frequency f propagating in the x-direction with wavenumber k we set the potentials to $\Phi(x,y,t) = \mathrm{Re}\{\phi(y)\exp[\mathrm{i}(kx - 2\pi f t)]\}$ and $\Psi(x,y,t) = \mathrm{Re}\{\psi(y)\exp[\mathrm{i}(kx - 2\pi f t)]\}$, obtaining from Eq. (1) the following system of equations

$$\frac{d^2\phi}{dy^2} + r^2\phi = 0, \tag{5a}$$

$$\frac{d^2\psi}{dy^2} + s^2\psi = 0, \tag{5b}$$

where $r^2 = k_\mathrm{l}^2 - k^2$, $s^2 = k_\mathrm{t}^2 - k^2$, $k_\mathrm{l}^2 = (2\pi f/c_\mathrm{l})^2$, $k_\mathrm{t}^2 = (2\pi f/c_\mathrm{t})^2$, $c_\mathrm{l}^2 = (\lambda + 2\mu)/\rho$ is the longitudinal wave speed and $c_\mathrm{t}^2 = \mu/\rho$ is the shear wave speed. The solution of Eqs. (5a,5b) is readily obtained as the superposition of harmonic waves

$$\begin{aligned} \phi(y) &= \phi^+(y)\mathrm{e}^{\mathrm{i}ry} + \phi^-(y)\mathrm{e}^{-\mathrm{i}ry}, \\ \psi(y) &= \psi^+(y)\mathrm{e}^{\mathrm{i}sy} + \psi^-(y)\mathrm{e}^{-\mathrm{i}sy}, \end{aligned} \tag{6}$$

where the signs + and − denote forward and backward propagation of GLWs in the y-direction. With reference to Fig.1(c) of the main paper, in the absence of corrugation (i.e. $2\epsilon = 0$) we set the traction-free boundary conditions $\sigma_{yy,xy} = 0$ in Eq. (4) at the planes $y_\pm = \pm h$, with $2h$ being the structural thickness. This leads to a homogeneous algebraic system of equations for the amplitudes $\psi^\pm, \phi^\pm$

$$(k^2 - s^2)\mathrm{e}^{\mathrm{i}sh}\psi^+ + (k^2 - s^2)\mathrm{e}^{-\mathrm{i}sh}\psi^- - 2rk\mathrm{e}^{\mathrm{i}rh}\phi^+ + 2rk\mathrm{e}^{-\mathrm{i}rh}\phi^- = 0, \tag{7a}$$

$$(k^2 - s^2)\mathrm{e}^{-\mathrm{i}sh}\psi^+ + (k^2 - s^2)\mathrm{e}^{\mathrm{i}sh}\psi^- - 2rk\mathrm{e}^{-\mathrm{i}rh}\phi^+ + 2rk\mathrm{e}^{\mathrm{i}rh}\phi^- = 0, \tag{7b}$$

$$2sk\mu\mathrm{e}^{\mathrm{i}sh}\psi^+ - 2sk\mu\mathrm{e}^{-\mathrm{i}sh}\psi^- - (\lambda k_\mathrm{l}^2 + 2\mu r^2)\mathrm{e}^{\mathrm{i}rh}\phi^+ - (\lambda k_\mathrm{l}^2 + 2\mu r^2)\mathrm{e}^{-\mathrm{i}rh}\phi^- = 0, \tag{7c}$$

$$2sk\mu\mathrm{e}^{-\mathrm{i}sh}\psi^+ - 2sk\mu\mathrm{e}^{\mathrm{i}sh}\psi^- - (\lambda k_\mathrm{l}^2 + 2\mu r^2)\mathrm{e}^{-\mathrm{i}rh}\phi^+ - (\lambda k_\mathrm{l}^2 + 2\mu r^2)\mathrm{e}^{\mathrm{i}rh}\phi^- = 0, \tag{7d}$$

which provides two well-known independent dispersion relations for both symmetric and antisymmetric modes

$$\frac{\tan(sh)}{\tan(rh)} = -\frac{4rsk^2}{(2k^2 - k_\mathrm{t}^2)^2} \qquad \text{(sym)}, \tag{8a}$$

$$\frac{\tan(sh)}{\tan(rh)} = -\frac{(2k^2 - k_\mathrm{t}^2)^2}{4rsk^2} \qquad \text{(antisym)}, \tag{8b}$$

and the corresponding eigenmodes

$$\begin{cases} \psi^+ = 1 \\ \psi^- = -1 \\ \phi^+ = \dfrac{(k^2 - s^2)\sin(sh)}{2rk\sin(rh)} \\ \phi^- = \dfrac{(k^2 - s^2)\sin(sh)}{2rk\sin(rh)} \end{cases} \text{(sym)} \ , \qquad \begin{cases} \phi^+ = 1 \\ \phi^- = -1 \\ \psi^+ = \dfrac{2rk\sin(rh)}{(k^2 - s^2)\cos(sh)} \\ \psi^- = \dfrac{2rk\sin(rh)}{(k^2 - s^2)\cos(sh)} \end{cases} \text{(antisym)} \ . \tag{9}$$

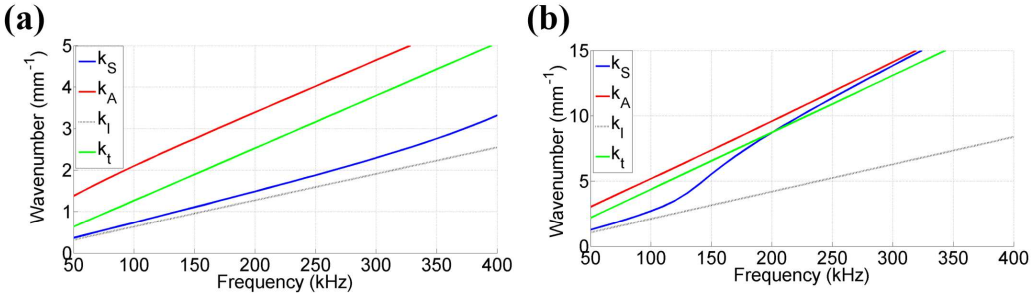

FIG. S1: Dispersion relations of k_{S}, k_{A}, k_{l}, and k_{t} for (**a**) aluminium and (**b**) ABS plate-like structures with thickness $2h = 10$ mm.

The displacement components u_x and u_y in Eq. (4) for both symmetric and antisymmetric modes can thus be expressed as

$$\begin{cases} u_x^{\mathrm{S}} = 2\mathrm{i}s\left[\cos(sy) + \dfrac{(k^2-s^2)}{2sr}\dfrac{\sin(sh)}{\sin(rh)}\cos(ry)\right]\mathrm{e}^{\mathrm{i}(kx-2\pi ft)} \\ u_y^{\mathrm{S}} = 2k\left[\sin(sy) - \dfrac{(k^2-s^2)}{2k^2}\dfrac{\sin(sh)}{\sin(rh)}\sin(ry)\right]\mathrm{e}^{\mathrm{i}(kx-2\pi ft)} \end{cases} \quad (\mathrm{sym}),$$
$$\begin{cases} u_x^{\mathrm{A}} = -2k\left[\sin(ry) + \dfrac{2sr}{(k^2-s^2)}\dfrac{\cos(rh)}{\cos(sh)}\sin(sy)\right]\mathrm{e}^{\mathrm{i}(kx-2\pi ft)} \\ u_y^{\mathrm{A}} = -2\mathrm{i}r\left[-\cos(ry) + \dfrac{2k^2}{(k^2-s^2)}\dfrac{\cos(rh)}{\cos(sh)}\cos(sy)\right]\mathrm{e}^{\mathrm{i}(kx-2\pi ft)} \end{cases} \quad (\mathrm{antisym})\,. \tag{10}$$

Fig.S1 illustrates the dispersion relations obtained from Eqs. (8a,8b) of the lowest symmetric and antisymmetric modes $k_{\mathrm{S,A}}$, the non-dispersive longitudinal and shear $k_{\mathrm{l,t}}$ modes of an uncorrugated plate of thickness $2h = 10$ mm and composed of either aluminium or acrylonitrile butadiene styrene (ABS) polymer.

II. ADIABATIC COUPLED-MODE THEORY FOR CORRUGATED WAVEGUIDES

In the presence of corrugation, we assume that the unperturbed symmetric and antisymmetric modes follow adiabatically the corrugated plate profile, so that we can study the perturbation of the lowest symmetric S_0 and antisymmetric A_0 waves induced by the corrugation [see Fig. 1(c) of the main paper]. Similarly to [3], the plate boundaries are described by the new conditions $y_{\pm} = \pm h - \epsilon\cos(2\pi x/\Lambda)$, so that the x, y-dependent symmetric $\mathbf{u}_{\mathrm{S}}^{\pm}$ and antisymmetric $\mathbf{u}_{\mathrm{A}}^{\pm}$ mode profiles of forward and backward GLWs are given by

$$\begin{aligned} \mathbf{u}_{\mathrm{S}}^{\pm} &= \begin{cases} 2\mathrm{i}s_{\mathrm{S}}\left\{\cos\left[s_{\mathrm{S}}(\tilde{y})\right] + \frac{(k_{\mathrm{S}}^2-s_{\mathrm{S}}^2)}{2s_{\mathrm{S}}r_{\mathrm{S}}}\frac{\sin(s_{\mathrm{S}}h)}{\sin(r_{\mathrm{S}}h)}\cos\left[r_{\mathrm{S}}(\tilde{y})\right]\right\} \\ \mp 2k_{\mathrm{S}}\left\{\sin\left[s_{\mathrm{S}}(\tilde{y})\right] - \frac{(k_{\mathrm{S}}^2-s_{\mathrm{S}}^2)}{2k_{\mathrm{S}}^2}\frac{\sin(s_{\mathrm{S}}h)}{\sin(r_{\mathrm{S}}h)}\sin\left[r_{\mathrm{S}}(\tilde{y})\right]\right\} \end{cases}, \\ \mathbf{u}_{\mathrm{A}}^{\pm} &= \begin{cases} \mp 2k_{\mathrm{A}}\left\{\sin\left[r_{\mathrm{A}}(\tilde{y})\right] + \frac{2s_{\mathrm{A}}r_{\mathrm{A}}}{(k_{\mathrm{A}}^2-s_{\mathrm{A}}^2)}\frac{\cos(r_{\mathrm{A}}h)}{\cos(s_{\mathrm{A}}h)}\sin\left[s_{\mathrm{A}}(\tilde{y})\right]\right\} \\ -2\mathrm{i}r_{\mathrm{A}}\left\{-\cos\left[r_{\mathrm{A}}(\tilde{y})\right] + \frac{2k_{\mathrm{A}}^2}{(k_{\mathrm{A}}^2-s_{\mathrm{A}}^2)}\frac{\cos(r_{\mathrm{A}}h)}{\cos(s_{\mathrm{A}}h)}\cos\left[s_{\mathrm{A}}(\tilde{y})\right]\right\} \end{cases}, \end{aligned} \tag{11}$$

where $\tilde{y} = y - \epsilon\cos(2\pi x/\Lambda)$, $s_{\mathrm{S,A}}^2 = k_{\mathrm{t}}^2 - k_{\mathrm{S,A}}^2$ and $r_{\mathrm{S,A}}^2 = k_{\mathrm{l}}^2 - k_{\mathrm{S,A}}^2$. Under the assumption $\epsilon \ll h$ the corrugation can be treated as perturbation, and the general solution $\mathbf{u}(x, y)$ of the GLW problem induced by the corrugation is

$$\mathbf{u}(x,y) = a_{\mathrm{S}}^{+}(x)\mathbf{u}_{\mathrm{S}}^{+}\mathrm{e}^{\mathrm{i}(k_{\mathrm{S}}x-2\pi ft)} + a_{\mathrm{S}}^{-}(x)\mathbf{u}_{\mathrm{S}}^{-}\mathrm{e}^{-\mathrm{i}(k_{\mathrm{S}}x+2\pi ft)} + a_{\mathrm{A}}^{+}(x)\mathbf{u}_{\mathrm{A}}^{+}\mathrm{e}^{\mathrm{i}(k_{\mathrm{A}}x-2\pi ft)} + a_{\mathrm{A}}^{-}(x)\mathbf{u}_{\mathrm{A}}^{-}\mathrm{e}^{-\mathrm{i}(k_{\mathrm{A}}x+2\pi ft)}, \tag{12}$$

where $a_{\mathrm{S,A}}^{\pm}$ represent the scalar amplitudes of symmetric and antisymmetric modes of the corrugated plate, and $\mathbf{u}_{\mathrm{S,A}}^{\pm}$ are defined in Eq. (11). Inserting the Ansatz of Eq. (12) into Eq. (1), and expanding the resulting system of

differential equations in multiple scales at the first order in the parameter ϵ/h, we achieve

$$
\begin{aligned}
&\frac{\partial a_{\mathrm{S}}^{+}}{\partial x}\begin{pmatrix} 2\mathrm{i}k_{\mathrm{S}}c_{\mathrm{l}}^{2} & (c_{\mathrm{l}}^{2}-c_{\mathrm{t}}^{2})\frac{\partial}{\partial y} \\ (c_{\mathrm{l}}^{2}-c_{\mathrm{t}}^{2})\frac{\partial}{\partial y} & 2\mathrm{i}k_{\mathrm{S}}c_{\mathrm{t}}^{2}\end{pmatrix}\mathbf{u}_{\mathrm{S}}^{+}\mathrm{e}^{\mathrm{i}(k_{\mathrm{S}}x-2\pi ft)} \\
&+a_{\mathrm{S}}^{+}\left(\frac{2\pi\epsilon}{\Lambda}\right)\sin\left(\frac{2\pi x}{\Lambda}\right)\begin{pmatrix} 2\mathrm{i}k_{\mathrm{S}}c_{\mathrm{l}}^{2} & (c_{\mathrm{l}}^{2}-c_{\mathrm{t}}^{2})\frac{\partial}{\partial y} \\ (c_{\mathrm{l}}^{2}-c_{\mathrm{t}}^{2})\frac{\partial}{\partial y} & 2\mathrm{i}k_{\mathrm{S}}c_{\mathrm{t}}^{2}\end{pmatrix}\frac{\partial\mathbf{u}_{\mathrm{S}}^{+}}{\partial y}\mathrm{e}^{\mathrm{i}(k_{\mathrm{S}}x-2\pi ft)} \\
&+a_{\mathrm{S}}^{+}\frac{4\pi^{2}\epsilon}{\Lambda^{2}}\cos\left(\frac{2\pi x}{\Lambda}\right)\begin{pmatrix} c_{\mathrm{l}}^{2} & 0 \\ 0 & c_{\mathrm{t}}^{2}\end{pmatrix}\frac{\partial\mathbf{u}_{\mathrm{S}}^{+}}{\partial y}\mathrm{e}^{\mathrm{i}(k_{\mathrm{S}}x-2\pi ft)} \\
&+\frac{\partial a_{\mathrm{S}}^{-}}{\partial x}\begin{pmatrix} -2\mathrm{i}k_{\mathrm{S}}c_{\mathrm{l}}^{2} & (c_{\mathrm{l}}^{2}-c_{\mathrm{t}}^{2})\frac{\partial}{\partial y} \\ (c_{\mathrm{l}}^{2}-c_{\mathrm{t}}^{2})\frac{\partial}{\partial y} & -2\mathrm{i}k_{\mathrm{S}}c_{\mathrm{t}}^{2}\end{pmatrix}\mathbf{u}_{\mathrm{S}}^{-}\mathrm{e}^{-\mathrm{i}(k_{\mathrm{S}}x+2\pi ft)} \\
&+a_{\mathrm{S}}^{-}\left(\frac{2\pi\epsilon}{\Lambda}\right)\sin\left(\frac{2\pi x}{\Lambda}\right)\begin{pmatrix} -2\mathrm{i}k_{\mathrm{S}}c_{\mathrm{l}}^{2} & (c_{\mathrm{l}}^{2}-c_{\mathrm{t}}^{2})\frac{\partial}{\partial y} \\ (c_{\mathrm{l}}^{2}-c_{\mathrm{t}}^{2})\frac{\partial}{\partial y} & -2\mathrm{i}k_{\mathrm{S}}c_{\mathrm{t}}^{2}\end{pmatrix}\frac{\partial\mathbf{u}_{\mathrm{S}}^{-}}{\partial y}\mathrm{e}^{-\mathrm{i}(k_{\mathrm{S}}x+2\pi ft)} \\
&+a_{\mathrm{S}}^{-}\frac{4\pi^{2}\epsilon}{\Lambda^{2}}\cos\left(\frac{2\pi x}{\Lambda}\right)\begin{pmatrix} c_{\mathrm{l}}^{2} & 0 \\ 0 & c_{\mathrm{t}}^{2}\end{pmatrix}\frac{\partial\mathbf{u}_{\mathrm{S}}^{-}}{\partial y}\mathrm{e}^{-\mathrm{i}(k_{\mathrm{S}}x+2\pi ft)} \\
&+\frac{\partial a_{\mathrm{A}}^{+}}{\partial x}\begin{pmatrix} 2\mathrm{i}k_{\mathrm{A}}c_{\mathrm{l}}^{2} & (c_{\mathrm{l}}^{2}-c_{\mathrm{t}}^{2})\frac{\partial}{\partial y} \\ (c_{\mathrm{l}}^{2}-c_{\mathrm{t}}^{2})\frac{\partial}{\partial y} & 2\mathrm{i}k_{\mathrm{A}}c_{\mathrm{t}}^{2}\end{pmatrix}\mathbf{u}_{\mathrm{A}}^{+}\mathrm{e}^{\mathrm{i}(k_{\mathrm{A}}x-2\pi ft)} \\
&+a_{\mathrm{A}}^{+}\left(\frac{2\pi\epsilon}{\Lambda}\right)\sin\left(\frac{2\pi x}{\Lambda}\right)\begin{pmatrix} 2\mathrm{i}k_{\mathrm{A}}c_{\mathrm{l}}^{2} & (c_{\mathrm{l}}^{2}-c_{\mathrm{t}}^{2})\frac{\partial}{\partial y} \\ (c_{\mathrm{l}}^{2}-c_{\mathrm{t}}^{2})\frac{\partial}{\partial y} & 2\mathrm{i}k_{\mathrm{A}}c_{\mathrm{t}}^{2}\end{pmatrix}\frac{\partial\mathbf{u}_{\mathrm{A}}^{+}}{\partial y}\mathrm{e}^{\mathrm{i}(k_{\mathrm{A}}x-2\pi ft)} \\
&+a_{\mathrm{A}}^{+}\frac{4\pi^{2}\epsilon}{\Lambda^{2}}\cos\left(\frac{2\pi x}{\Lambda}\right)\begin{pmatrix} c_{\mathrm{l}}^{2} & 0 \\ 0 & c_{\mathrm{t}}^{2}\end{pmatrix}\frac{\partial\mathbf{u}_{\mathrm{A}}^{+}}{\partial y}\mathrm{e}^{\mathrm{i}(k_{\mathrm{A}}x-2\pi ft)} \\
&+\frac{\partial a_{\mathrm{A}}^{-}}{\partial x}\begin{pmatrix} -2\mathrm{i}k_{\mathrm{A}}c_{\mathrm{l}}^{2} & (c_{\mathrm{l}}^{2}-c_{\mathrm{t}}^{2})\frac{\partial}{\partial y} \\ (c_{\mathrm{l}}^{2}-c_{\mathrm{t}}^{2})\frac{\partial}{\partial y} & -2\mathrm{i}k_{\mathrm{A}}c_{\mathrm{t}}^{2}\end{pmatrix}\mathbf{u}_{\mathrm{A}}^{-}\mathrm{e}^{-\mathrm{i}(k_{\mathrm{A}}x+2\pi ft)} \\
&+a_{\mathrm{A}}^{-}\left(\frac{2\pi\epsilon}{\Lambda}\right)\sin\left(\frac{2\pi x}{\Lambda}\right)\begin{pmatrix} -2\mathrm{i}k_{\mathrm{A}}c_{\mathrm{l}}^{2} & (c_{\mathrm{l}}^{2}-c_{\mathrm{t}}^{2})\frac{\partial}{\partial y} \\ (c_{\mathrm{l}}^{2}-c_{\mathrm{t}}^{2})\frac{\partial}{\partial y} & -2\mathrm{i}k_{\mathrm{A}}c_{\mathrm{t}}^{2}\end{pmatrix}\frac{\partial\mathbf{u}_{\mathrm{A}}^{-}}{\partial y}\mathrm{e}^{-\mathrm{i}(k_{\mathrm{A}}x+2\pi ft)} \\
&+a_{\mathrm{A}}^{-}\frac{4\pi^{2}\epsilon}{\Lambda^{2}}\cos\left(\frac{2\pi x}{\Lambda}\right)\begin{pmatrix} c_{\mathrm{l}}^{2} & 0 \\ 0 & c_{\mathrm{t}}^{2}\end{pmatrix}\frac{\partial\mathbf{u}_{\mathrm{A}}^{-}}{\partial y}\mathrm{e}^{-\mathrm{i}(k_{\mathrm{A}}x+2\pi ft)}=0,
\end{aligned}
\tag{13}
$$

where we have neglected second order derivatives ∂_x^2 by assuming them of the order of $\mathrm{o}(\epsilon/h)^2$. Thus, in order to get the solvability condition of Eqs. (13), we take the scalar product with unperturbed symmetric/antisymmetric forward/backward modes [Eq. (10)] in the limit of vanishing corrugation. Doing so, we find that the couplings between forward and backward modes of identical symmetry turns out to vanish, while couplings between forward and backward modes of opposite symmetries remain finite. This leads to the following system of coupled differential equations for the mode amplitudes

$$\frac{da_{\mathrm{S}}^{+}(x)}{dx}=a_{\mathrm{A}}^{-}(x)C_{\mathrm{S}}\left(\frac{\pi\epsilon}{\Lambda}\right)\mathrm{e}^{\mathrm{i}\left[\frac{2\pi}{\Lambda}-(k_{\mathrm{A}}+k_{\mathrm{S}})\right]x}, \tag{14a}$$

$$\frac{da_{\mathrm{S}}^{-}(x)}{dx}=a_{\mathrm{A}}^{+}(x)C_{\mathrm{S}}\left(\frac{\pi\epsilon}{\Lambda}\right)\mathrm{e}^{-\mathrm{i}\left[\frac{2\pi}{\Lambda}-(k_{\mathrm{A}}+k_{\mathrm{S}})\right]x}, \tag{14b}$$

$$\frac{da_{\mathrm{A}}^{+}(x)}{dx}=a_{\mathrm{S}}^{-}(x)C_{\mathrm{S}}\left(\frac{\pi\epsilon}{\Lambda}\right)\mathrm{e}^{\mathrm{i}\left[\frac{2\pi}{\Lambda}-(k_{\mathrm{A}}+k_{\mathrm{S}})\right]x}, \tag{14c}$$

$$\frac{da_{\mathrm{A}}^{-}(x)}{dx}=a_{\mathrm{S}}^{+}(x)C_{\mathrm{S}}\left(\frac{\pi\epsilon}{\Lambda}\right)\mathrm{e}^{-\mathrm{i}\left[\frac{2\pi}{\Lambda}-(k_{\mathrm{A}}+k_{\mathrm{S}})\right]x}, \tag{14d}$$

where $C_\mathrm{S} = -(C_1 - \mathrm{i}C_2)/C_3$, $C_\mathrm{A} = -(C_4 - \mathrm{i}C_5)/C_6$, and

$$
\begin{aligned}
C_1 &= \left(\frac{2\pi}{\Lambda}\right)\int_{-h}^{h} dy \left[\mathbf{u}_\mathrm{S}^{+*}\cdot\begin{pmatrix} c_\mathrm{l}^2 & 0 \\ 0 & c_\mathrm{t}^2\end{pmatrix}\frac{\partial \mathbf{u}_\mathrm{A}^-}{\partial y}\right] = -\left(\frac{2\pi}{\Lambda}\right)\int_{-h}^{h} dy \left[\mathbf{u}_\mathrm{S}^{-*}\cdot\begin{pmatrix} c_\mathrm{l}^2 & 0 \\ 0 & c_\mathrm{t}^2\end{pmatrix}\frac{\partial \mathbf{u}_\mathrm{A}^+}{\partial y}\right], \\
C_2 &= \int_{-h}^{h} dy \left[\mathbf{u}_\mathrm{S}^{+*}\cdot\begin{pmatrix} -2\mathrm{i}k_\mathrm{A}c_\mathrm{l}^2 & (cl^2 - c_\mathrm{t}^2)\frac{\partial}{\partial y} \\ (cl^2 - c_\mathrm{t}^2)\frac{\partial}{\partial y} & -2\mathrm{i}k_\mathrm{A}c_\mathrm{t}^2\end{pmatrix}\frac{\partial \mathbf{u}_\mathrm{A}^-}{\partial y}\right] = \int_{-h}^{h} dy \left[\mathbf{u}_\mathrm{S}^{-*}\cdot\begin{pmatrix} 2\mathrm{i}k_\mathrm{A}c_\mathrm{l}^2 & (cl^2 - c_\mathrm{t}^2)\frac{\partial}{\partial y} \\ (cl^2 - c_\mathrm{t}^2)\frac{\partial}{\partial y} & 2\mathrm{i}k_\mathrm{A}c_\mathrm{t}^2\end{pmatrix}\frac{\partial \mathbf{u}_\mathrm{A}^+}{\partial y}\right], \\
C_3 &= \int_{-h}^{h} dy \left[\mathbf{u}_\mathrm{S}^{+*}\cdot\begin{pmatrix} 2\mathrm{i}k_\mathrm{S}c_\mathrm{l}^2 & (cl^2 - c_\mathrm{t}^2)\frac{\partial}{\partial y} \\ (cl^2 - c_\mathrm{t}^2)\frac{\partial}{\partial y} & 2\mathrm{i}k_\mathrm{S}c_\mathrm{t}^2\end{pmatrix}\mathbf{u}_\mathrm{S}^+\right] = -\int_{-h}^{h} dy \left[\mathbf{u}_\mathrm{S}^{-*}\cdot\begin{pmatrix} -2\mathrm{i}k_\mathrm{S}c_\mathrm{l}^2 & (cl^2 - c_\mathrm{t}^2)\frac{\partial}{\partial y} \\ (cl^2 - c_\mathrm{t}^2)\frac{\partial}{\partial y} & -2\mathrm{i}k_\mathrm{S}c_\mathrm{t}^2\end{pmatrix}\mathbf{u}_\mathrm{S}^-\right], \\
C_4 &= \int_{-h}^{h} dy \left[\mathbf{u}_\mathrm{A}^{+*}\cdot\begin{pmatrix} 2\mathrm{i}k_\mathrm{A}c_\mathrm{l}^2 & (cl^2 - c_\mathrm{t}^2)\frac{\partial}{\partial y} \\ (cl^2 - c_\mathrm{t}^2)\frac{\partial}{\partial y} & 2\mathrm{i}k_\mathrm{A}c_\mathrm{t}^2\end{pmatrix}\mathbf{u}_\mathrm{A}^+\right] = -\int_{-h}^{h} dy \left[\mathbf{u}_\mathrm{A}^{-*}\cdot\begin{pmatrix} -2\mathrm{i}k_\mathrm{A}c_\mathrm{l}^2 & (cl^2 - c_\mathrm{t}^2)\frac{\partial}{\partial y} \\ (cl^2 - c_\mathrm{t}^2)\frac{\partial}{\partial y} & -2\mathrm{i}k_\mathrm{A}c_\mathrm{t}^2\end{pmatrix}\mathbf{u}_\mathrm{A}^-\right], \\
C_5 &= \left(\frac{2\pi}{\Lambda}\right)\int_{-h}^{h} dy \left[\mathbf{u}_\mathrm{A}^{+*}\cdot\begin{pmatrix} c_\mathrm{l}^2 & 0 \\ 0 & c_\mathrm{t}^2\end{pmatrix}\frac{\partial \mathbf{u}_\mathrm{S}^-}{\partial y}\right] = -\left(\frac{2\pi}{\Lambda}\right)\int_{-h}^{h} dy \left[\mathbf{u}_\mathrm{A}^{-*}\cdot\begin{pmatrix} c_\mathrm{l}^2 & 0 \\ 0 & c_\mathrm{t}^2\end{pmatrix}\frac{\partial \mathbf{u}_\mathrm{S}^+}{\partial y}\right], \\
C_6 &= \int_{-h}^{h} dy \left[\mathbf{u}_\mathrm{A}^{+*}\cdot\begin{pmatrix} -2\mathrm{i}k_\mathrm{S}c_\mathrm{l}^2 & (cl^2 - c_\mathrm{t}^2)\frac{\partial}{\partial y} \\ (cl^2 - c_\mathrm{t}^2)\frac{\partial}{\partial y} & -2\mathrm{i}k_\mathrm{S}c_\mathrm{t}^2\end{pmatrix}\frac{\partial \mathbf{u}_\mathrm{S}^-}{\partial y}\right] = \int_{-h}^{h} dy \left[\mathbf{u}_\mathrm{S}^{-*}\cdot\begin{pmatrix} 2\mathrm{i}k_\mathrm{S}c_\mathrm{l}^2 & (cl^2 - c_\mathrm{t}^2)\frac{\partial}{\partial y} \\ (cl^2 - c_\mathrm{t}^2)\frac{\partial}{\partial y} & 2\mathrm{i}k_\mathrm{S}c_\mathrm{t}^2\end{pmatrix}\frac{\partial \mathbf{u}_\mathrm{S}^+}{\partial y}\right].
\end{aligned}
\tag{15}
$$

The propagation of forward and backward modes accounted by Eqs. (14a-14d) can be uncoupled into two second-order dispersion equations for the forward propagating modes

$$\frac{d^2 a_\mathrm{S}^+(x)}{dx^2} = \mathrm{i}\left[\frac{2\pi}{\Lambda} - (k_\mathrm{A} + k_\mathrm{S})\right]\frac{d a_\mathrm{S}^+(x)}{dx} + C_\mathrm{S}C_\mathrm{A}\left(\frac{\pi\epsilon}{\Lambda}\right)^2 a_\mathrm{S}^+(x), \tag{16a}$$

$$\frac{d^2 a_\mathrm{A}^+(x)}{dx^2} = \mathrm{i}\left[\frac{2\pi}{\Lambda} - (k_\mathrm{A} + k_\mathrm{S})\right]\frac{d a_\mathrm{A}^+(x)}{dx} + C_\mathrm{S}C_\mathrm{A}\left(\frac{\pi\epsilon}{\Lambda}\right)^2 a_\mathrm{A}^+(x), \tag{16b}$$

which provide the solutions $a_\mathrm{S}^+(x) = A_\mathrm{S}^+ e^{\mathrm{i}\beta x}$ and $a_\mathrm{A}^+(x) = A_\mathrm{A}^+ e^{\mathrm{i}\beta x}$, where the corrugation-induced wavenumber shift β is

$$\beta = \frac{1}{2}\left[\frac{2\pi}{\Lambda} - (k_\mathrm{A} + k_\mathrm{S})\right] + \frac{1}{2}\sqrt{\left[\frac{2\pi}{\Lambda} - (k_\mathrm{A} + k_\mathrm{S})\right]^2 - 4C_\mathrm{S}C_\mathrm{A}\left(\frac{\pi\epsilon}{\Lambda}\right)^2}. \tag{17}$$

Note that for the forward mode solution we neglected a second solution for the wavenumber shift β, which reflects the propagation of backward modes. The coupling coefficients (15) are explicitly given by

$$
\begin{aligned}
C_1 &= \left(\frac{2\pi}{\Lambda}\right)\int_{-h}^{h} dy \left[\begin{array}{l}
-4\mathrm{i}s_\mathrm{S}^* k_\mathrm{A} c_\mathrm{l}^2 \left\{ \frac{2s_\mathrm{A}^2 r_\mathrm{A}}{\left(k_\mathrm{A}^2-s_\mathrm{A}^2\right)} \frac{\cos(r_\mathrm{A}h)}{\cos(s_\mathrm{A}h)} \cos(s_\mathrm{A}y) + r_\mathrm{A}\cos(r_\mathrm{A}y) \right\} \\
\left\{ \frac{\left(k_\mathrm{S}^2-s_\mathrm{S}^{2*}\right)}{2s_\mathrm{S}^* r_\mathrm{S}^*} \frac{\sin(s_\mathrm{S}^*h)}{\sin(r_\mathrm{S}^*h)} \cos(r_\mathrm{S}^*y) + \cos(s_\mathrm{S}^*y) \right\} \\
+4\mathrm{i}r_\mathrm{A} k_\mathrm{S} c_\mathrm{t}^2 \left\{ \frac{2s_\mathrm{A} k_\mathrm{A}^2}{\left(k_\mathrm{A}^2-s_\mathrm{A}^2\right)} \frac{\cos(r_\mathrm{A}h)}{\cos(s_\mathrm{A}h)} \sin(s_\mathrm{A}y) - r_\mathrm{A}\sin(r_\mathrm{A}y) \right\} \\
\left\{ -\frac{\left(k_\mathrm{S}^2-s_\mathrm{S}^{2*}\right)}{2k_\mathrm{S}^2} \frac{\sin(s_\mathrm{S}^*h)}{\sin(r_\mathrm{S}^*h)} \sin(r_\mathrm{S}^*y) + \sin(s_\mathrm{S}^*y) \right\}
\end{array}\right], \\
C_2 &= \int_{-h}^{h} dy \left[\begin{array}{l}
-4r_\mathrm{A} s_\mathrm{S}^* \left\{ \frac{\left(k_\mathrm{S}^2-s_\mathrm{S}^{2*}\right)}{2s_\mathrm{S}^* r_\mathrm{S}^*} \frac{\sin(s_\mathrm{S}^*h)}{\sin(r_\mathrm{S}^*h)} \cos(r_\mathrm{S}^*y) + \cos(s_\mathrm{S}^*y) \right\} \\
\left\{ \frac{2s_\mathrm{A}^2 k_\mathrm{A}^2}{\left(k_\mathrm{A}^2-s_\mathrm{A}^2\right)} (c_\mathrm{l}^2+c_\mathrm{t}^2) \frac{\cos(r_\mathrm{A}h)}{\cos(s_\mathrm{A}h)} \cos(s_\mathrm{A}y) + \left[r_\mathrm{A}^2(c_\mathrm{l}^2-c_\mathrm{t}^2) + 2k_\mathrm{A}^2 c_\mathrm{l}^2\right]\cos(r_\mathrm{A}y) \right\} \\
+4k_\mathrm{S} k_\mathrm{A} r_\mathrm{A} \left\{ -\frac{\left(k_\mathrm{S}^2-s_\mathrm{S}^{2*}\right)}{2k_\mathrm{S}^2} \frac{\sin(s_\mathrm{S}^*h)}{\sin(r_\mathrm{S}^*h)} \sin(r_\mathrm{S}^*y) + \sin(s_\mathrm{S}^*y) \right\} \\
\left\{ \frac{2s_\mathrm{A}}{\left(k_\mathrm{A}^2-s_\mathrm{A}^2\right)} \left[2c_\mathrm{t}^2 k_\mathrm{A}^2 - \left(c_\mathrm{l}^2-c_\mathrm{t}^2\right)s_\mathrm{A}^2\right] \frac{\cos(r_\mathrm{A}h)}{\cos(s_\mathrm{A}h)} \sin(s_\mathrm{A}y) - r_\mathrm{A}(c_\mathrm{l}^2+c_\mathrm{t}^2)\sin(r_\mathrm{A}y) \right\}
\end{array}\right], \\
C_3 &= \int_{-h}^{h} dy \left[\begin{array}{l}
2\mathrm{i}s_\mathrm{S}^* \left\{ \frac{\left(k_\mathrm{S}^2-s_\mathrm{S}^{2*}\right)}{2s_\mathrm{S}^* r_\mathrm{S}^*} \frac{\sin(s_\mathrm{S}^*h)}{\sin(r_\mathrm{S}^*h)} \cos(r_\mathrm{S}^*y) + \cos(s_\mathrm{S}^*y) \right\} \\
\left\{ 2k_\mathrm{S} s_\mathrm{S}\left(c_\mathrm{l}^2+c_\mathrm{t}^2\right)\cos(s_\mathrm{S}y) + \left[\left(c_\mathrm{l}^2-c_\mathrm{t}^2\right)\frac{r_\mathrm{S}}{k_\mathrm{S}} + 2cl^2\frac{k_\mathrm{S}}{r_\mathrm{S}}\right]\left(k_\mathrm{S}^2-s_\mathrm{S}^2\right)\frac{\sin(s_\mathrm{S}h)}{\sin(r_\mathrm{S}h)}\cos(r_\mathrm{S}y) \right\} \\
+2\mathrm{i}k_\mathrm{S} \left\{ -\frac{\left(k_\mathrm{S}^2-s_\mathrm{S}^{2*}\right)}{2k_\mathrm{S}^2} \frac{\sin(s_\mathrm{S}^*h)}{\sin(r_\mathrm{S}^*h)} \sin(r_\mathrm{S}^*y) + \sin(s_\mathrm{S}^*y) \right\} \\
\left\{ 2\left[2k_\mathrm{S}^2 c_\mathrm{t}^2 - s_\mathrm{S}^2\left(c_\mathrm{l}^2-c_\mathrm{t}^2\right)\right]\sin(s_\mathrm{S}y) - \left(k_\mathrm{S}^2-s_\mathrm{S}^2\right)\left(c_\mathrm{l}^2+c_\mathrm{t}^2\right)\frac{\sin(s_\mathrm{S}h)}{\sin(r_\mathrm{S}h)}\sin(r_\mathrm{S}y) \right\}
\end{array}\right], \\
C_4 &= \int_{-h}^{h} dy \left[\begin{array}{l}
4\mathrm{i}k_\mathrm{A} \left\{ \frac{2s_\mathrm{A}^* r_\mathrm{A}^*}{\left(k_\mathrm{A}^2-s_\mathrm{A}^{2*}\right)} \frac{\cos(r_\mathrm{A}^*h)}{\cos(s_\mathrm{A}^*h)} \sin(s_\mathrm{A}^*y) + \sin(r_\mathrm{A}^*y) \right\} \\
\left\{ \frac{2s_\mathrm{A} r_\mathrm{A} k_\mathrm{A}^2}{\left(k_\mathrm{A}^2-s_\mathrm{A}^2\right)} \left(c_\mathrm{l}^2+c_\mathrm{t}^2\right) \frac{\cos(r_\mathrm{A}h)}{\cos(s_\mathrm{A}h)} \sin(s_\mathrm{A}y) + \left[r_\mathrm{A}^2\left(c_\mathrm{l}^2-c_\mathrm{t}^2\right) + 2k_\mathrm{A}^2 c_\mathrm{l}^2\right]\sin(r_\mathrm{A}y) \right\} \\
+4\mathrm{i}k_\mathrm{A} r_\mathrm{A}^2 \left\{ \frac{2k_\mathrm{A}^2}{\left(k_\mathrm{A}^2-s_\mathrm{A}^{2*}\right)} \frac{\cos(r_\mathrm{A}^*h)}{\cos(s_\mathrm{A}^*h)} \cos(s_\mathrm{A}^*y) - \cos(r_\mathrm{A}^*y) \right\} \\
\left\{ \frac{2\left[2k_\mathrm{A}^2 c_\mathrm{t}^2 - \left(c_\mathrm{l}^2-c_\mathrm{t}^2\right)s_\mathrm{A}^2\right]}{\left(k_\mathrm{A}^2-s_\mathrm{A}^2\right)} \frac{\cos(r_\mathrm{A}h)}{\cos(s_\mathrm{A}h)} \cos(s_\mathrm{A}y) - \left(c_\mathrm{l}^2+c_\mathrm{t}^2\right)\cos(r_\mathrm{A}y) \right\}
\end{array}\right], \\
C_5 &= \left(\frac{2\pi}{\Lambda}\right)\int_{-h}^{h} dy \left[\begin{array}{l}
2\mathrm{i}k_\mathrm{A} c_\mathrm{l}^2 \left\{ 2s_\mathrm{S}^2\sin(s_\mathrm{S}y) + \left(k_\mathrm{S}^2-s_\mathrm{S}^2\right)\frac{\sin(s_\mathrm{S}h)}{\sin(r_\mathrm{S}h)}\sin(r_\mathrm{S}y) \right\} \\
\left\{ \frac{2s_\mathrm{A}^* r_\mathrm{A}^*}{\left(k_\mathrm{A}^2-s_\mathrm{A}^{2*}\right)} \frac{\cos(r_\mathrm{A}^*h)}{\cos(s_\mathrm{A}^*h)} \sin(s_\mathrm{A}^*y) + \sin(r_\mathrm{A}^*y) \right\} \\
-2\mathrm{i}r_\mathrm{A}^* c_\mathrm{t}^2 \left\{ 2k_\mathrm{S} s_\mathrm{S}\cos(s_\mathrm{S}y) - r_\mathrm{S}\frac{\left(k_\mathrm{S}^2-s_\mathrm{S}^2\right)}{k_\mathrm{S}}\frac{\sin(s_\mathrm{S}h)}{\sin(r_\mathrm{S}h)}\sin(r_\mathrm{S}y) \right\} \\
\left\{ \frac{2k_\mathrm{A}^2}{\left(k_\mathrm{A}^2-s_\mathrm{A}^{2*}\right)} \frac{\cos(r_\mathrm{A}^*h)}{\cos(s_\mathrm{A}^*h)} \cos(s_\mathrm{A}^*y) - \cos(r_\mathrm{A}^*y) \right\}
\end{array}\right], \\
C_6 &= \int_{-h}^{h} dy \left[\begin{array}{l}
2k_\mathrm{A} \left\{ \frac{2s_\mathrm{A}^* r_\mathrm{A}^*}{\left(k_\mathrm{A}^2-s_\mathrm{A}^{2*}\right)} \frac{\cos(r_\mathrm{A}^*h)}{\cos(s_\mathrm{A}^*h)} \sin(s_\mathrm{A}^*y) + \sin(r_\mathrm{A}^*y) \right\} \\
\left\{ 2k_\mathrm{S} s_\mathrm{S}^2\left(c_\mathrm{l}^2+c_\mathrm{t}^2\right)\sin(s_\mathrm{S}y) + \left(k_\mathrm{S}^2-s_\mathrm{S}^2\right)\left[\frac{r_\mathrm{S}^2}{k_\mathrm{S}}\left(c_\mathrm{l}^2-c_\mathrm{t}^2\right) + 2k_\mathrm{S} c_\mathrm{l}^2\right]\frac{\sin(s_\mathrm{S}h)}{\sin(r_\mathrm{S}h)}\sin(r_\mathrm{S}y) \right\} \\
+2r_\mathrm{A}^* \left\{ \frac{2k_\mathrm{A}^2}{\left(k_\mathrm{A}^2-s_\mathrm{A}^{2*}\right)} \frac{\cos(r_\mathrm{A}^*h)}{\cos(s_\mathrm{A}^*h)} \cos(s_\mathrm{A}^*y) - \cos(r_\mathrm{A}^*y) \right\} \\
\left\{ 2s_\mathrm{S}\left[s_\mathrm{S}^2\left(c_\mathrm{l}^2-c_\mathrm{t}^2\right) - 2k_\mathrm{S}^2 c_\mathrm{t}^2\right]\cos(s_\mathrm{S}y) + r_\mathrm{S}\left(k_\mathrm{S}^2-s_\mathrm{S}^2\right)\left(c_\mathrm{l}^2+c_\mathrm{t}^2\right)\frac{\sin(s_\mathrm{S}h)}{\sin(r_\mathrm{S}h)}\cos(r_\mathrm{S}y) \right\}
\end{array}\right],
\end{aligned}
\tag{18}
$$

where the sign $(*)$ denotes the complex conjugate operation. We used the wavenumber shift β analytically provided by Eq. (17) to design our PC transducers. Fig.S2 illustrates the analytical results of the real and imaginary parts of the corrugation-induced wavenumber shift $\beta = \beta' + \mathrm{i}\beta''$ for both aluminium ($\Lambda = 8.1$ mm) and ABS ($\Lambda = 2.1$ mm) PC waveguides with thickness $2h = 10$ mm. As reported in the paper, we used a corrugation depth of $2\epsilon = 3$ mm.

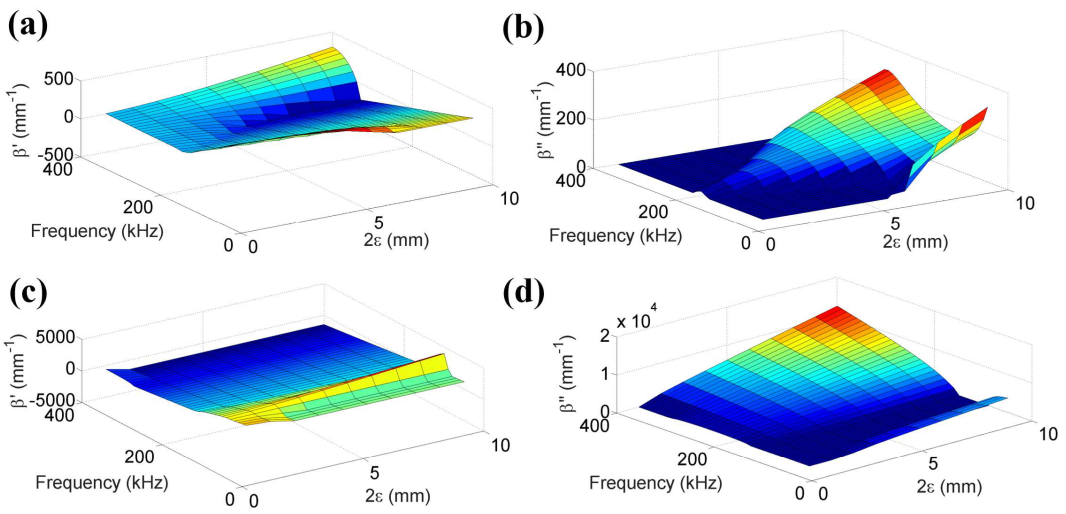

FIG. S2: Analytical results of the real and imaginary parts of β as function of frequency f and corrugation depth 2ϵ for PC waveguides with average thickness $2h = 10$ mm. (**a,c**) Real and (**b,d**) imaginary parts of the corrugation-induced wavenumber shift β for (**a,b**) aluminium PCs with $\Lambda = 8.1$ mm and (**c,d**) ABS PCs with $\Lambda = 2.1$ mm.

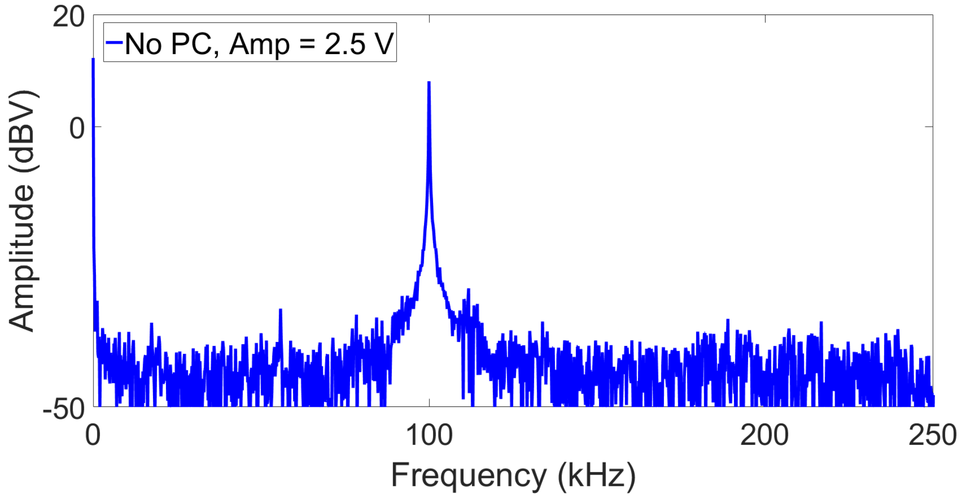

FIG. S3: Experimental ultrasonic response measured in the absence of PC structure and amplification. The input voltage is 2.5 V.

III. EXPERIMENTAL ULTRASONIC RESULTS IN THE ABSENCE OF AMPLIFICATION

As reported in the Introduction section of the paper, the input voltage in nonlinear ultrasonic experiments usually ranges between 50 V and 150 V. This is generally due to the material attenuation of the transmitted signal and the quadratic dependence of the amplitude of the second harmonic frequency over the fundamental one. The maximum input amplitude of current signal generators is typically 5-10 V peak-to-peak, thus pre-amplification systems are necessary. No pre-amplification of the transmitted signal would inevitably result in a low amplitude fundamental frequency with the associated second harmonic frequency hidden into the noise. As an example, Fig.S3 illustrates the material response measured by the receiver transducer in the absence of pre-amplification system and with no PC waveguide using an input voltage of 2.5 V. Such a value of input voltage was chosen for comparison with Figure 4(b) of the manuscript as it corresponds to the original amplitude before pre-amplification. Indeed, since our pre-amplification system provides a gain of 50, by applying 2.5 V as input, the voltage amplifier is able to deliver 125 V that is the voltage used to perform the nonlinear ultrasonic experiments in Figure 4(b).

From Fig.S3, it can be seen that at the input voltage of 2.5 V, the amplitude of the fundamental frequency $f_0 =$ 100 kHz is 7.8 dBV [compared to 45 dBV at 125 V in Figure 4(b)], whereas, as expected, the second harmonic

amplitude at $2f_0 = 200$ kHz is very low and hidden into the noise.

---

[1] Rose, J.L. and Nagy, P.B. Ultrasonic waves in solid media. *The Journal of the Acoustical Society of America* **107**(4), 1807-1808 (2000).
[2] Viktorov, I.A. Rayleigh and Lamb Waves: Physical Theory and Applications (New York, Plenum press, 1967).
[3] Asfar, O., Hawwa, M., Bavencoffe, M., Morvan, B., and Izbicki, J. L. A multiple-scale perturbation approach to mode coupling in periodic plates. *IEEE transactions on ultrasonics, ferroelectrics, and frequency control*, **60**(2), 395-401 (2013).
